# Nivolumab Enhances the Cytotoxicity of Chemotherapeutic Agents in A549 Lung Adenocarcinoma Cell Lines

**DOI:** 10.3390/cimb48050443

**Published:** 2026-04-24

**Authors:** Nilgün Okşak, Oğur Karhan

**Affiliations:** 1 Faculty of Health Science, Harran University, Şanlıurfa 63300, Turkey; nilgunoksak@harran.edu.tr; 2 Department of Medical Oncology, Faculty of Medicine, Harran University Hospital, Şanlıurfa 63300, Turkey

**Keywords:** nivolumab, cytotoxicity, chemotherapy, lung adenocarcinoma cell line

## Abstract

Background and Objectives: The integration of chemotherapy (ChT) and immune checkpoint inhibitors (ICIs) has become a standard approach in oncology. Although the addition of ICIs to double-agent ChT regimens has demonstrated clinical benefit in multiple studies, other trials have reported no significant improvement. ChT is hypothesized to potentiate the effects of ICIs through multiple mechanisms, including tumor antigen release and modulation of the tumor microenvironment. This study aimed to evaluate whether nivolumab enhances the cytotoxic effects of cisplatin or paclitaxel in lung adenocarcinoma (A549) cell lines under immune-independent conditions. Materials and Methods: A549 lung alveolar carcinoma cell lines were treated with varying concentrations of nivolumab, cisplatin, and paclitaxel, individually and in combinations. Cytotoxicity and apoptosis were assessed using mitochondrial membrane potential analysis, cell viability assays, and morphological evaluation of cellular and nuclear alterations characteristic of apoptotic cell death. Results: Nivolumab alone exhibited no cytotoxic activity. The combination of cisplatin at its IC_50_ (half-maximal inhibitory concentration) (3 µg/mL) with 13 µg/mL nivolumab yielded the most pronounced cytotoxicity (89%) compared to cisplatin alone (49%, *p* < 0.001). Paclitaxel combined with nivolumab increased cytotoxicity to 69% versus 51% for paclitaxel alone (*p* < 0.05). The enhancement effect was greater with cisplatin than with paclitaxel. Notably, adding nivolumab to the cisplatin–paclitaxel combination reduced cytotoxicity from 73% to 64%. Mechanistic analysis revealed a significant reduction in Rhodamine 123 fluorescence intensity in drug-treated groups versus controls (*p* < 0.001), indicating loss of mitochondrial membrane potential, a hallmark of intrinsic apoptotic activation, suggesting apoptotic priming. Conclusions: Nivolumab potentiates the cytotoxic effects of cisplatin and paclitaxel in A549 lung adenocarcinoma cells, with a more pronounced effect observed in combination with cisplatin. This enhancement is associated with mitochondrial membrane potential loss, supporting mitochondrial apoptotic priming as a potential underlying mechanism of drug synergy.

## 1. Introduction

Immunotherapy, particularly immune checkpoint inhibitors (ICIs), has revolutionized the therapeutic landscape across multiple malignancies, including lung cancer [1,2,3]. Historically, chemotherapy (ChT) has served as the cornerstone of lung cancer treatment [4]. The integration of ICIs with platinum-based doublet chemotherapy has demonstrated significant clinical benefit [5]. Notably, the addition of nivolumab and ipilimumab to ChT has been shown to improve median progression-free survival (mPFS) from 10.9 months to 15.6 months [4]. Similarly, the combination of pembrolizumab with doublet ChT has resulted in an increase in mPFS from 10.6 months to 22 months in the management of metastatic non-small cell lung cancer (NSCLC) [6].

ChT may potentiate the efficacy of ICIs by promoting neoantigen expression, enhancing immune cell infiltration, activating natural killer cells, and reducing immunosuppressive cell populations [7]. However, the immunomodulatory effects of chemotherapy agents are not uniform. For instance, cisplatin has been shown to upregulate genes involved in the T-cell cytotoxicity pathway [8], while paclitaxel promotes the infiltration of cytotoxic T lymphocytes (CTLs). Both cisplatin and paclitaxel sensitize tumor cells to CTLs by increasing their permeability to granzyme B, thereby enhancing tumor cell susceptibility to immune-mediated cytotoxicity [7].

Despite the potential synergy, concerns remain that ChT may induce lymphopenia and impair T-cell function, potentially diminishing the efficacy of ICIs [9]. The addition of ICIs to ChT does not universally enhance clinical outcomes. For example, the combination of pembrolizumab with ChT failed to improve outcomes in gastric cancer [10]. Similarly, the addition of atezolizumab to ChT demonstrated no significant benefit in patients with advanced triple-negative breast cancer [11].

These data underscore persistent questions regarding the optimal integration of ICIs with ChT in cancer treatment. Key considerations include whether ICIs should be combined with doublet chemotherapy or whether reducing ChT to monotherapy could enhance cytotoxic effects. While preclinical and clinical evidence indicate that ChT enhances ICI efficacy, it remains unclear whether ICIs reciprocally enhance ChT effectiveness.

To investigate these questions, in this study, lung adenocarcinoma (A549) cell lines were treated with cisplatin, paclitaxel and nivolumab, either alone or in various combinations. The aim of this study was to determine the most effective regimen and to assess whether ICIs can enhance the efficacy of chemotherapy in vitro. The assessment of cytotoxic and apoptotic effects was conducted through the utilization of morphological and biochemical methods. These encompassed the evaluation of mitochondrial membrane potential, cell viability, and morphological changes in cells and nuclei.

## 2. Materials and Methods

### 2.1. Cell Culture

The A549 cell line was obtained from the American Type Culture Collection (ATCC) (Manassas, VA, USA). Cells were cultured in Dulbecco’s Modified Eagle Medium (DMEM; Biowest, Nuaillé, France, B.L0102-500) supplemented with 10% fetal bovine serum (FBS; HYCLONE, South American, SV30160.03), 1% penicillin/streptomycin (P/S; HYCLONE, SV30010), and 1% glutamine. Cultures were maintained at 37 °C in a humidified atmosphere containing 5% CO_2_. The culture medium was replenished every 2–3 days, and cells were passaged upon reaching 80–90% confluence.

### 2.2. Cytotoxicity Analysis

The cytotoxic effects of the drugs were assessed using the 3-(4,5-dimethylthiazol-2-yl)-2,5-diphenyltetrazolium bromide (MTT) assay (Roche, Basel, Switzerland, M2003-1G). A549 cells were seeded in 96-well flat-bottom plates at a density of 10^4^ cells per well. Cells were allowed to adhere for approximately two hours prior to treatment. Subsequently, cells were exposed to cisplatin (Koçak Pharma, Bağcılar/İstanbul, Turkey), paclitaxel (Koçak Pharma, Turkey), nivolumab (Bristol-Myers Squibb, New York, NY, USA), and various combinations of these agents. Treated cells were incubated for 48 h at 37 °C in a humidified environment with 5% CO_2_.

Following a 48-h incubation, 10 μL of MTT (5 mg/mL) was added to each well and incubated for an additional 4 h. At the end of the incubation period, 100 μL of DMSO (Neofroxx 1084ML100, Einhausen, Germany) was added to dissolve the formazan crystals. Optical density (OD) was measured at 570 nm, with a reference wavelength of 630 nm, using an ELISA reader (MD Spectramax M5, San Jose, CA, USA). Cell viability was calculated by comparing the absorbance of treated wells to control wells.

The half-maximal inhibitory concentration (IC_50_) values were determined to assess cytotoxicity. IC_50_ represents the concentration required to inhibit 50% of cell growth. IC_50_ values were calculated for cisplatin (0.75–10 μg/mL), paclitaxel (0.3–2.4 μg/mL), and their various combinations (Table 1). Nivolumab was administered at doses of 13, 30, and 50 μg/mL.

### 2.3. Assessment of Apoptotic Activity

Apoptotic activity was evaluated by assessing mitochondrial membrane potential and performing morphological analysis through DAPI (4′,6-diamidino-2-phenylindole) staining.

#### 2.3.1. Measurement of Mitochondrial Membrane Potential

Mitochondrial membrane potential (Δψm) was assessed to evaluate early apoptotic events. The measurement of Δψm correlates with the accumulation of fluorescent dye within mitochondria, providing a quantitative assessment of mitochondrial integrity [12]. Rhodamine 123 (Rho 123) selectively stains mitochondria, allowing for differentiation between viable and damaged cells based on fluorescence intensity. Lower fluorescence signals indicate mitochondrial dysfunction and cellular damage [13].

Experimental and control group cells were seeded at a density of 10^5^ cells/mL in 24-well plates and incubated in a humidified incubator (Heal Force, HF 212 UV, Shanghai, China) at 37 °C with 5% CO_2_ for 48 h. The experimental groups received the concentrations listed in Table 1. Following incubation, cells were washed twice with 100 μL of PBS. Fixation was performed using a 3% glutaraldehyde buffer (MERCK, Darmstadt, Germany, 8206031000) for one hour, followed by additional PBS washes. Cells were then incubated with 10 μM rhodamine 123 (Glentham, Corsham, UK, GT3585-5MG) for 30 min at 37 °C in a 5% CO_2_ incubator. After staining, cells were washed three times with ice-cold PBS (Thermo, Waltham, MA, USA, 10010023) to remove excess dye. Rho 123 accumulation was visualized using fluorescence microscopy and quantified using ImageJ software 1.54g. For each image, 20 fields were analyzed, and the mean fluorescence intensity, along with the standard deviation, was calculated.

#### 2.3.2. Morphological Assessment of Cell Apoptosis

Morphological evaluation of apoptosis in control and experimental groups was conducted using light microscopy and DAPI fluorescence staining. After 48 h of treatment, nuclear morphology was analyzed in response to drug concentrations outlined in Table 1. DAPI staining revealed chromatin condensation and fragmentation characteristics of apoptotic cells. A549 cells were seeded at a density of 10^5^ cells/mL in 24-well plates and incubated in a humidified environment at 37 °C with 5% CO_2_ for 48 h. Following treatment, cells were fixed with a 3% glutaraldehyde buffer for one hour and washed with PBS. DAPI staining (1 μg; Abbkine, Wuhan, China, BMD00063) was performed by incubating the cells for 15 min at 37 °C in a 5% CO_2_ atmosphere. After washing, cells were visualized using an Olympus EX51 epifluorescence microscope (Tokyo, Japan) to assess apoptotic nuclear morphology.

### 2.4. Statistical Analysis

All data were analyzed using one-way analysis of variance (ANOVA) and expressed as the mean ± standard deviation (SD) from three independent experiments (n = 3). Statistical comparisons between groups and controls were performed using GraphPad Prism 5.04. A *p*-value of less than 0.05 (*p* < 0.05) was considered statistically significant.

## 3. Results

### 3.1. Cytotoxicity Analysis

**Figure 1:**
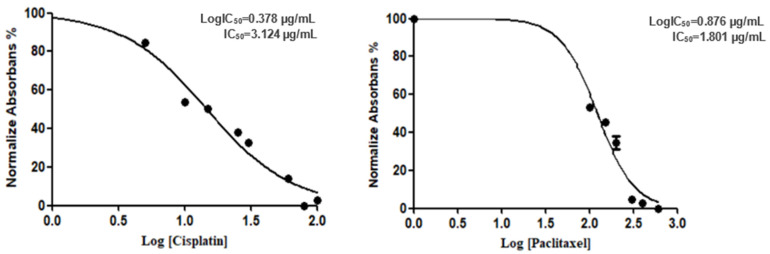
IC_50_ values for cisplatin and paclitaxel in A549 cells.

This study aimed to evaluate the effects of cisplatin, paclitaxel, nivolumab, and their combinations on the viability of the human alveolar adenocarcinoma A549 cell line. Cytotoxicity was assessed by determining the IC_50_ values following a 48-h treatment under conditions consistent with the MTT assay. Initial experiments established the IC_50_ values for cisplatin and paclitaxel. The IC_50_ for cisplatin was determined to be 3.124 µg/mL (Log IC_50_: 0.378 µg/mL), while the IC_50_ for paclitaxel was 1.801 µg/mL (LogIC_50_: 0.876 µg/mL), as depicted in Figure 1.

Nivolumab did not exhibit direct cytotoxic effects within the tested concentration range (13, 30, and 50 µg/mL). When applied to A549 cells, cell viability remained relatively stable at 94%, 90%, and 101% for concentrations of 13, 30, and 50 µg/mL, respectively.

**Figure 2:**
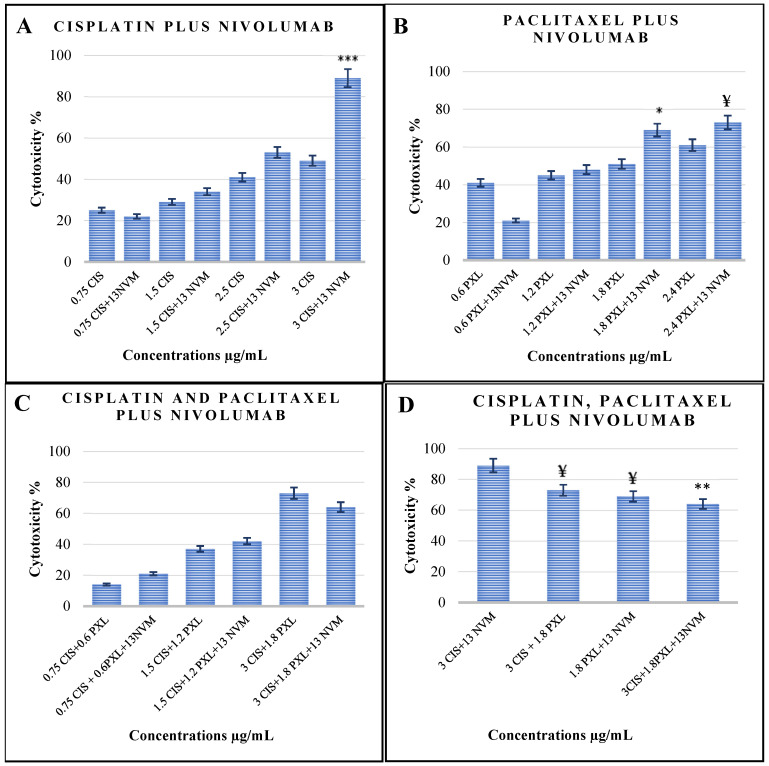
Cytotoxicity values in the A549 cell line treated with Cisplatin (CIS), Paclitaxel (PXL), and Nivolumab (NVM), and their combinations. (**A**) In the cisplatin and combination treatment groups, a significant increase in cytotoxicity was observed. *** *p* < 0.001 for 3 µg/mL CIS + 13 µg/mL NVM compared to 3 µg/mL CIS alone. (**B**) In the paclitaxel and combination treatment groups, the addition of nivolumab significantly enhanced cytotoxicity. * *p* < 0.05 for 1.8 µg/mL PXL + 13 µg/mL NVM compared to 1.8 µg/mL PXL alone, and ^¥^ *p* < 0.05 for 2.4 µg/mL PXL + 13 µg/mL NVM compared to 2.4 µg/mL PXL alone. (**C**) In triple-drug combination groups (cisplatin, paclitaxel, and nivolumab), cytotoxicity was analyzed across various dose combinations. (**D**) Double-drug and triple-drug combinations were compared. ** *p* < 0.01 for 3 µg/mL CIS + 1.8 µg/mL PXL + 13 µg/mL NVM compared to 3 µg/mL CIS + 13 µg/mL NVM. Additionally, ^¥^ *p* < 0.05 for 3 µg/mL CIS + 1.8 µg/mL PXL compared to 3 µg/mL CIS + 13 µg/mL NVM and for 1.8 µg/mL PXL + 13 µg/mL NVM compared to 3 µg/mL CIS + 13 µg/mL NVM. Differences between groups were analyzed using one-way ANOVA (n = 3).

The cytotoxic effects of varying concentrations of nivolumab in combination with cisplatin or paclitaxel were evaluated. As illustrated in Figure 2A, four different concentrations of cisplatin and paclitaxel were tested in combination with 13 µg/mL nivolumab. The most pronounced combined cytotoxic effect was observed in the group treated with 3 µg/mL cisplatin plus 13 µg/mL nivolumab, achieving a cytotoxicity of 89%, compared to 49% with cisplatin alone (Figure 2A, *p* < 0.001). In contrast, at lower doses of cisplatin, the addition of nivolumab resulted in only modest increases in cytotoxicity. For example, 0.75 µg/mL cisplatin alone demonstrated 25% cytotoxicity, while the combination of 13 µg/mL nivolumab with 0.75 µg/mL cisplatin resulted in 22% cytotoxicity. Similarly, 1.5 µg/mL cisplatin alone exhibited 29% cytotoxicity, compared to 34% with the addition of 13 µg/mL nivolumab (Figure 2A).

The cytotoxic effect of the combination of paclitaxel and nivolumab showed minimal changes when low doses of paclitaxel were combined with 13 µg/mL nivolumab. For instance, at a paclitaxel dose of 1.2 µg/mL, the addition of 13 µg/mL nivolumab resulted in a slight increase in cytotoxicity, from 45% to 48%. Conversely, at the lowest paclitaxel dose (0.6 µg/mL), the combination decreased cytotoxicity from 41% to 21% compared to paclitaxel alone. At higher doses of paclitaxel, the combination showed more pronounced effects. Specifically, the combination of 2.4 µg/mL paclitaxel and 13 µg/mL nivolumab achieved 73% cytotoxicity, compared to 61% with paclitaxel alone (Figure 2B, *p* < 0.05).

As illustrated in Figure 2C, an enhanced cytotoxic effect was observed when nivolumab was added to low-dose combinations of two anticancer agents. For example, 0.75 µg/mL cisplatin combined with 0.6 µg/mL paclitaxel resulted in 14% cytotoxicity, which increased to 21% upon the addition of 13 µg/mL nivolumab. Similarly, the addition of 13 µg/mL nivolumab to 1.5 µg/mL cisplatin and 1.2 µg/mL paclitaxel increased cytotoxicity from 37% to 42%. In contrast, at higher chemotherapy doses, the addition of nivolumab reduced cytotoxicity. A combination of 3 µg/mL cisplatin and 1.8 µg/mL paclitaxel demonstrated 73% cytotoxicity, which decreased to 64% when 13 µg/mL nivolumab was added to the regimen.

We compared the cytotoxic effects of double-drug and triple-drug combinations. The combination of 3 µg/mL cisplatin and 13 µg/mL nivolumab demonstrated the highest cytotoxicity at 89%. Substituting 13 µg/mL nivolumab with 1.8 µg/mL paclitaxel reduced the cytotoxicity to 73%. Furthermore, the addition of 1.8 µg/mL paclitaxel to the combination of 3 µg/mL cisplatin and 13 µg/mL nivolumab decreased the cytotoxicity from 89% to 64% (Figure 2D, *p* < 0.05). The results for the cytotoxic activity of the different combinations of cisplatin, paclitaxel, and nivolumab were also presented as a table in the Supplementary Materials (Table S1).

Table 1 shows the doses of cisplatin, paclitaxel, and nivolumab that, when given together to A549 cells, caused 50% of the cells to die. According to the table, the IC_50_ values for cisplatin and paclitaxel were determined to be 3 μg/mL and 1.8 μg/mL, respectively. Cell viability was comparable when 13 µg/mL nivolumab was added to a lower dosage of cisplatin (2.5 µg/mL) or paclitaxel (1.2 µg/mL). Similarly, it was shown that a 50% cell death rate required a combination of 1.2 μg/mL paclitaxel and 2.5 μg/mL cisplatin. Nevertheless, a 50% cell death rate was also observed when paclitaxel was excluded and 13 μg/mL nivolumab was added to cisplatin. 50% of the cells died when 2 µg/mL cisplatin was added to 1.2 µg/mL paclitaxel and 13 µg/mL nivolumab in the triple combination. The concentrations at which 50% inhibition of cell viability was observed are summarized in Table 1.

### 3.2. Assessment of Apoptotic Activity

#### 3.2.1. Measurement of Mitochondrial Membrane Potential

The mean Rho 123 fluorescence intensity (MFI) values for A549 cells are summarized in Table 2. In untreated A549 cells (control), the mean fluorescence intensity was 53.7 ± 8.5. Drug-treated groups showed a significant reduction in MFI, with mean values ranging from 9.0 ± 2.3 to 27.3 ± 7.5 (*p* < 0.001).

**Figure 3:**
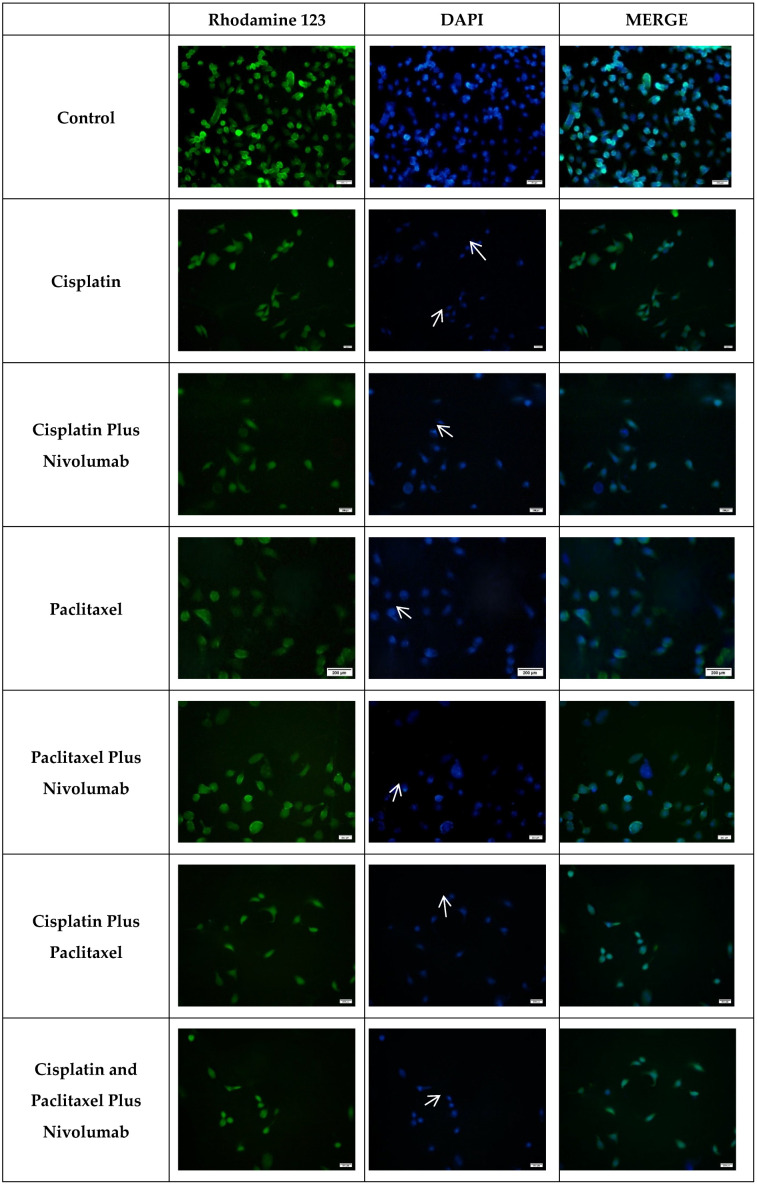
Rhodamine 123 and DAPI staining images of A549 cells treated with drug combinations of cisplatin, paclitaxel, and nivolumab. The control group consists of A549 cells that have not been treated with any drug. The concentrations shown in Table 1 were used. The scale bar is 100 µm, and the magnification is ×20.

Figure 3 illustrates the Rhodamine 123 fluorescence staining images of control and drug-treated A549 cells. The fluorescence intensity of Rho 123 was markedly higher in the untreated control group, as evidenced by brighter fluorescence signals. In contrast, the drug-treated groups exhibited a noticeable reduction in fluorescence intensity, appearing dimmer by comparison, indicative of decreased mitochondrial membrane potential.

#### 3.2.2. Morphological Assessment of Cell Apoptosis

DNA fragmentation, a hallmark of apoptosis, was evaluated using DAPI staining, a widely utilized and reliable technique for identifying apoptotic cells [14,15]. The DAPI staining analysis provided significant insights into the apoptosis of A549 cells treated with cisplatin, paclitaxel, and nivolumab.

**Figure 4:**
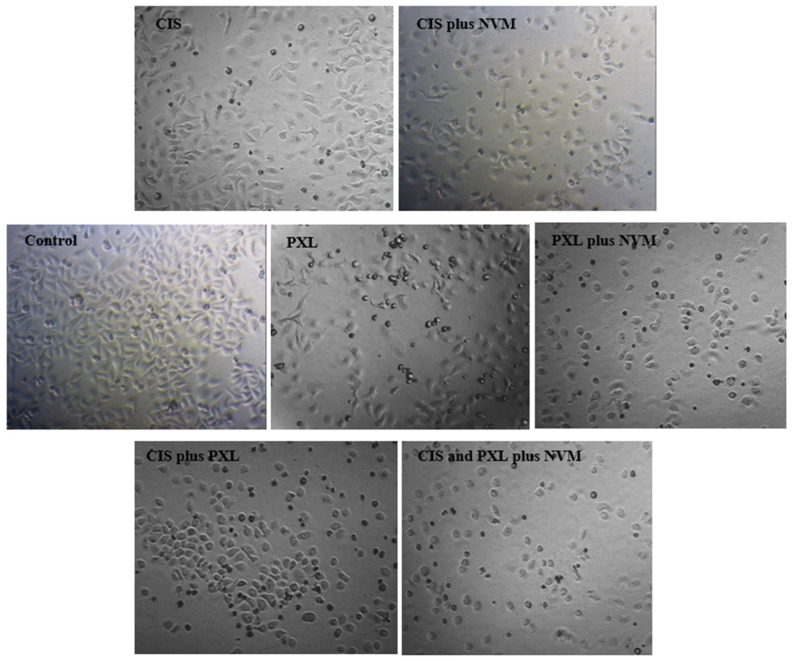
Light microscopy images of control and experimental groups. Control: Untreated A549 cells; CIS: cisplatin-treated cells; NVM: nivolumab-treated cells; PXL: paclitaxel-treated cells. The doses administered to the groups are equivalent to those administered to the groups specified in Table 1. ×60 magnification.

In untreated healthy cells, nuclei exhibited a characteristic intact morphology. In contrast, the nuclei of cells treated with cisplatin, paclitaxel, or nivolumab demonstrated clear apoptotic features, including nuclear shrinkage and chromatin condensation, resulting in bright, intense fluorescence within the nuclei. These cells are shown by white arrows (as shown in Figure 3). Additionally, morphological changes were observed in drug-treated lung cancer cells using light microscopy (Figure 4). Drug exposure led to a noticeable reduction in cell diameter and cell number. Non-adherent floating cells displayed a rounded morphology, in contrast to the adherent and elongated shape of untreated cells. These observations strongly suggest that the treated cells are undergoing apoptotic cell death.

## 4. Discussion

Immune checkpoint inhibitors, particularly anti-PD-1 agents (nivolumab and pembrolizumab), are presumed to block the PD-1 receptor on lymphocytes and induce lymphocyte activation, resulting in a tumoricidal effect. However, PD-L1 expressions do not reliably predict responsiveness to anti-PD-1 agents; even in certain cases, PD-L1-negative cancer patients respond to anti-PD-1/PD-L1 therapy. These observations raise questions regarding the mechanistic pathways of anti-PD-1/PD-L1 therapeutics [16]. Contrary to common assumptions, apart from predominant expression on T lymphocytes, PD-1 is intrinsically expressed in various cancers, including, but not limited to, melanoma, colorectal, and lung cancer [17,18,19]. Targeting of PD-1 on tumor cells mediates tumor regression through modulation of mTOR signaling in mouse models [17]. In our study, nivolumab exhibited no evidence of a direct cytotoxic effect on lung cancer cells in isolation; it was found to potentiate the cytotoxic effect of cisplatin, particularly when administered in combination. These results indicate that nivolumab can increase cytotoxic activity in the absence of the immune system. In contrast to our findings, one study reported that anti-PD-1 inhibition alone suppressed RT4 bladder cell proliferation [20]. This inconsistency between our study and the study mentioned above may stem from the different types of tumor cells used. However, the cytotoxic effect of anti-PD-1 agents in the absence of the immune system supports our findings.

In recent years, researchers have explored a variety of combination therapies and chemical agents to combat lethal diseases such as cancer and HIV. The primary objectives of these approaches are to achieve synergistic therapeutic effects, reduce drug dosages and associated toxicity, and minimize or delay the development of drug resistance, thereby maximizing the overall benefits of the treatment [21,22]. Accordingly, in the present study, the cytotoxic effect of cisplatin and paclitaxel combined with 13 µg/mL nivolumab using IC_50_ doses or lower was investigated. The IC_50_ values for cisplatin and paclitaxel were determined to be 3 μg/mL and 1.8 μg/mL, respectively.

In the context of combination drug regimens, the addition of 13 µg/mL nivolumab to 3 µg/mL cisplatin significantly increased cytotoxicity to 89%, compared to 50% cytotoxicity observed with 3 µg/mL cisplatin alone (*p* < 0.001). However, this effect diminished at lower doses of cisplatin. For instance, adding 13 µg/mL nivolumab to 1.5 µg/mL cisplatin only slightly increased cytotoxicity from 29% to 34%. Paclitaxel demonstrated similar, though not identical, results compared to cisplatin. When 13 µg/mL nivolumab was added to 1.8 µg/mL paclitaxel, cytotoxicity increased from 51% to 69% (*p* < 0.05). This 18% increase in cytotoxicity was notably lower than the 50% increase observed with the combination of cisplatin and nivolumab. These results indicate that while nivolumab exhibits a cytotoxic effect when combined with paclitaxel, this effect is less pronounced than when combined with cisplatin. This highlights that the cytotoxic effect of nivolumab varies depending on the chemotherapeutic agent it is combined with. To the best of our knowledge, there is limited in vitro research in the literature on the combined administration of nivolumab with cisplatin or paclitaxel. However, one study with a similar focus was identified, which evaluated the combined effects of several immune checkpoint inhibitors (ICIs) and chemotherapeutic agents. This study utilized both HCC-44 and A549 lung cancer cell lines and reported that, while durvalumab potentiated the effects of various chemotherapy agents, nivolumab reduced the effectiveness of chemotherapy, including cisplatin [23]. At first glance, these findings appear to contradict our results. Nevertheless, we hypothesise that the fundamental difference between this study and ours stems from the experimental design. In this study, a single dose of cisplatin and paclitaxel was used in combination with nivolumab. In contrast, in our study, the IC_50_ values for cisplatin and paclitaxel were first determined, and for the combination experiments, both IC_50_ and sub-IC_50_ doses were administered in combination with nivolumab (Figure 2). This highlights a critical finding: the extent of the cytotoxic effect is highly dependent on both the dose of the chemotherapeutic agent and its combination with nivolumab. In another study, cisplatin increased PD-1 expression on lung cancer cell lines, and anti-PD-1 antibody demonstrated antitumor activity in the absence of a lymphocyte-containing medium; its combination with cisplatin also exhibited a synergistic, stronger cytotoxic effect [24]. This study’s findings closely align with those of our study: the combination of cisplatin and nivolumab was observed to have a greater effect on cytotoxicity in a medium devoid of the immune system. In summary, these findings are particularly significant as they raise an important question regarding current clinical practices in medical oncology. ICIs are typically combined with double-agent chemotherapy regimens, such as cisplatin and paclitaxel. However, the results of this study suggest the need to explore whether monochemotherapy—specifically cisplatin in combination with an ICI—might be more effective than double-agent chemotherapy with an ICI.

In the triple drug combination, it was found that the viability of A549 cells was 50% when 2 μg/mL cisplatin, 1.2 μg/mL paclitaxel and 13 μg/mL nivolumab were administered together. It was also demonstrated that this viability value was achieved when nivolumab at 13 μg/mL was administered in combination with either 2.5 μg/mL cisplatin or 1.2 μg/mL paclitaxel. This result indicates that one chemotherapeutic agent can potentially be omitted and replaced with nivolumab, providing an alternative therapeutic strategy.

The mitochondrial membrane potential (ΔΨm) has been demonstrated to play a vital role in overall mitochondrial function [25]. Mitochondrial outer membrane permeability (MOMP) has been identified as a pivotal event that instigates apoptosis by facilitating the release of pro-apoptotic proteins from the intermembrane space (IMS) [26]. Chemotherapy is believed to primarily induce the death of cancer cells via the mitochondrial apoptotic pathway [27].

The mitochondrial apoptotic priming hypothesis posits that cancer cells exhibit augmented chemosensitivity, attributable to their elevated apoptotic priming, in comparison to normal cells [28]. One way of understanding this is through the measurement of ΔΨm, which is proportional to the accumulation of mitochondrial fluorescent dye [13]. Accordingly, our results showed a significant reduction in Rhodamine 123 fluorescence intensity in groups treated with anticancer drugs compared to control groups (*p* < 0.001; Table 2). This reduction indicates a loss of mitochondrial membrane potential, suggesting that drug-treated cells undergo apoptosis. We propose that the combination of nivolumab with ChT, particularly cisplatin, may be prime lung cancer cells for apoptosis more effectively than ChT alone.

Further evidence of apoptosis was observed through DAPI staining, which revealed nuclear shrinkage and a reduction in cell number. Bright fluorescence, indicative of chromatin condensation, was detected in apoptotic cells (Figure 3, white arrows). These data corroborate the concept that nivolumab augments the apoptotic priming of lung cancer cells when administered in conjunction with chemotherapy. It is also one of the results that suggests the morphological alterations seen in the cells are caused by apoptosis. It is also one of the results that suggests the morphological alterations seen in the cells are caused by apoptosis. According to our findings, while cells in the control group exhibited normal proliferation in a colony-like state—that is, they spread across the surface and interconnected—in cells treated with chemotherapeutic agents or a combination of these agents with nivolumab, a reduction in cell size (round cells indicated by white arrows) and detachment from the substrate were observed. Furthermore, the reductions in intercellular connections and cell count were noteworthy (Figure 4).

Our study has some limitations. Firstly, it was conducted on A549 lung adenocarcinoma cell lines, and therefore, the findings cannot be generalized to other cell lines. Additionally, the results observed in vitro may not necessarily translate into vivo conditions. Moreover, the findings of this study are specific to the combination of nivolumab with cisplatin and paclitaxel and cannot be generalized to other ICIs or chemotherapeutic agents. We believe our research sheds light on an underexplored aspect of chemoimmunotherapy, providing a foundation for further investigation.

## 5. Conclusions

In conclusion, nivolumab demonstrated no direct cytotoxicity on A549 lung adenocarcinoma cell lines cultured in media lacking immune system components. However, the addition of nivolumab at IC_50_ doses to cisplatin or paclitaxel treatment significantly enhanced cytotoxicity, with a more pronounced effect observed when combined with cisplatin. This cytotoxic effect diminished when nivolumab was added to the combination of cisplatin and paclitaxel. The observed cytotoxic effects of nivolumab and chemotherapeutics were associated with a decrease in mitochondrial membrane potential, suggesting that lung cancer cells become more prone to mitochondrial apoptotic priming.

## Figures and Tables

**Table 1:** Concentrations at which 50% inhibition of cell viability (IC_50_) was achieved for cisplatin, paclitaxel, and their combinations with nivolumab.

Treatment Group	Cisplatin Dose (µg/mL)	Paclitaxel Dose (µg/mL)	Nivolumab Dose (µg/mL)
**Cisplatin Alone**	3	-	-
**Paclitaxel Alone**	-	1.8	-
**Cisplatin + Nivolumab**	2.5	-	13
**Paclitaxel + Nivolumab**	-	1.2	13
**Cisplatin + Paclitaxel**	2.5	1.2	-
**Cisplatin + Paclitaxel + Nivolumab**	2	1.2	13

**Table 2:** MFI Values of Rhodamine 123 in Control (untreated with drugs) and Drug-Treated Groups. Means and standard deviations (X ± SD). Statistical significance (*p* < 0.001) was determined between the control group and Cisplatin (3 µg/mL) ^a^, Cisplatin (2.5 µg/mL) + Nivolumab (13 µg/mL) ^b^, Paclitaxel (1.8 µg/mL) ^c^, Paclitaxel (1.2 µg/mL) + Nivolumab (13 µg/mL) ^d^, Cisplatin (2.5 µg/mL) + Paclitaxel (1.2 µg/mL) ^e^, Cisplatin (2 µg/mL) + Paclitaxel (1.2 µg/mL) + Nivolumab (13 µg/mL) ^f^, Cisplatin (3 µg/mL) + Paclitaxel (1.8 µg/mL) ^g^, Cisplatin (3 µg/mL) + Nivolumab (13 µg/mL) ^ğ^, Paclitaxel (1.8 µg/mL) + Nivolumab (13 µg/mL) ^h^, and Cisplatin (3 µg/mL) + Paclitaxel (1.8 µg/mL) + Nivolumab (13 µg/mL) ^ı^.

Group	MFI X ± SD	Min–Max
Control	53.7 ± 8.5	39.3–68.0
Cisplatin (3 µg/mL)	24.5 ± 4.2 ^a^	16.8–32.7
Cisplatin (2.5 µg/mL) + Nivolumab (13 µg/mL)	22.3 ± 4.4 ^b^	15.4–32.1
Paclitaxel (1.8 µg/mL)	23.8 ± 5.0 ^c^	16.3–33.7
Paclitaxel (1.2 µg/mL) + Nivolumab (13 µg/mL)	27.2 ± 6.1 ^d^	18.2–37.8
Cisplatin (2.5 µg/mL) + Paclitaxel (1.2 µg/mL)	27.3 ± 7.5 ^e^	13.2–38.0
Cisplatin (2 µg/mL) + Paclitaxel (1.2 µg/mL) + Nivolumab (13 µg/mL)	24.6 ± 5.1 ^f^	15.1–34.5
Cisplatin (3 µg/mL) + Paclitaxel (1.8 µg/mL)	15.6 ± 5.8 ^g^	9.7–20.5
Cisplatin (3 µg/mL) + Nivolumab (13 µg/mL)	9.0 ± 2.3 ^ğ^	6.3–14.1
Paclitaxel (1.8 µg/mL) + Nivolumab (13 µg/mL)	19.4 ± 5.2 ^h^	10.6–32.4
Cisplatin (3 µg/mL) + Paclitaxel (1.8 µg/mL) + Nivolumab (13 µg/mL)	21.2 ± 7.1 ^ı^	16.1–37.7

## Data Availability

The original contributions presented in this study are included in the article/Supplementary Materials. Further inquiries can be directed to the corresponding author.

## Supplementary Materials

The following supporting information can be downloaded at: https://www.mdpi.com/article/10.3390/cimb48050443/s1.

## Abbreviations

The following abbreviations are used in this manuscript:

ICI	Immune checkpoint inhibitor
mPFS	Median progression-free survival
ATCC	American Type Culture Collection
ChT	Chemotherapy
ANOVA	One-way analysis of variance
DAPI	4′,6-diamidino-2-phenylindole
MTT	3-(4,5-dimethylthiazol-2-yl)-2,5-diphenyltetrazolium bromide
Rho 123	Rhodamine 123
SD	standard deviation
NSCLC	Non-small cell lung cancer
IC_50_	Half-maximal inhibitory concentration
CTL	Cytotoxic T lymphocyte
Δψm	Mitochondrial membrane potential
MFI	Mean Rho 123 fluorescence intensity
MOMP	Mitochondrial outer membrane permeabilization
IMS	Mitochondrial intermembrane space
