# Supplementary material for: Nivolumab Enhances the Cytotoxicity of Chemotherapeutic Agents in A549 Lung Adenocarcinoma Cell Lines
Source: Curr Issues Mol Biol. 2026 Apr 24;48(5):443. doi: 10.3390/cimb48050443 (PMC13204535; doi:10.3390/cimb48050443)
Supplement: Supplementary file 1 [file cimb-48-00443-s001.zip › cimb-4203062-supplementary.pdf]

Cytotoxicity (%) main results		Cytotoxicity (%) cisplatin combination	
Cisplatin (3 µg/mL)	49	Cisplatin (0.75 µg/mL)	25
Cisplatin (3 µg/mL)+nivolumab (13 µg/mL)	89	Cisplatin (0.75 µg/mL)+ nivolumab (13 µg/mL)	22
Paclitaxel (1.8 µg/mL)	51	Cisplatin (1.5 µg/mL)	29
Paclitaxel (1.8 µg/mL)+ nivolumab (13 µg/mL)	69	Cisplatin (1.5 µg/mL)+ nivolumab (13 µg/mL)	34
Cisplatin (3 µg/mL)+ Paclitaxel (1.8 µg/mL)	73	Cisplatin (2.5 µg/mL)	41
Cisplatin (3 µg/mL)+ Paclitaxel (1.8 µg/mL)+nivolumab (13 µg/mL)	64	Cisplatin (2.5 µg/mL)+ nivolumab (13 µg/mL)	53
**Cytotoxicity (%) paclitaxel combination**		**Cytotoxicity (%) triple combination**	
Paclitaxel (0.6 µg/mL)	41	Cisplatin (0.75 µg/mL)+ Paclitaxel (0.6 µg/mL)	14
Paclitaxel (0.6 µg/mL)+ nivolumab (13 µg/mL)	21	Cisplatin (0.75 µg/mL)+ Paclitaxel (0.6 µg/mL)+ nivolumab (13 µg/mL)	21
Paclitaxel (1.2 µg/mL)	45	Cisplatin (1.5 µg/mL)+ Paclitaxel (1.2 µg/mL)	37
Paclitaxel (1.2 µg/mL)+ nivolumab (13 µg/mL)	48	Cisplatin (1.5 µg/mL)+ Paclitaxel (1.2 µg/mL)+ nivolumab (13 µg/mL)	42
Paclitaxel (2.4 µg/mL)	61		
Paclitaxel (2.4 µg/mL)+ nivolumab (13 µg/mL)	73		

Table S1. Combined doses and cytotoxic effects of cisplatin, paclitaxel and nivolumab in A549 cells.

Commented [M1]: We added it

Commented [du2R1]: I confirm
